# Cancer evolution and heterogeneity

**DOI:** 10.1002/ags3.12182

**Published:** 2018-07-04

**Authors:** Koshi Mimori, Tomoko Saito, Atsushi Niida, Satoru Miyano

**Affiliations:** ^1^ Department of Surgery Kyushu University Beppu Hospital Beppu Japan; ^2^ Division of Health Medical Computational Science Health Intelligence Center Institute of Medical Science The University of Tokyo Tokyo Japan; ^3^ Laboratory of Sequence Analysis Human Genome Center Institute of Medical Science University of Tokyo Tokyo Japan

**Keywords:** cellular automaton, intratumor heterogeneity, The ratio between the rate of non‐synonymous substitutions per non‐synonymous site and the rate of synonymous substitutions per synonymous site, variant allele frequency, whole‐exome sequencing

## Abstract

Undoubtedly, intratumor heterogeneity (ITH) is one of the causes of the intractability of cancers. Recently, technological innovation in genomics has promoted studies on ITH in solid tumors and on the pattern and level of diversity, which varies among malignancies. We profiled the genome in multiple regions of nine colorectal cancer (CRC) cases. The most impressive finding was that in the late phase, a parental clone branched into numerous subclones. We found that minor mutations were dominant in advanced CRC named neutral evolution; that is, driver gene aberrations were observed with high proportion in the early‐acquired phase, but low in the late‐acquired phase. Then, we validated that neutral evolution could cause ITH in advanced CRC by super‐computational analysis. According to the clinical findings, we explored a branching evolutionary process model in cancer evolution, which assumes that each tumor cell has cellular automaton. According to the model, we verified factors to foster ITH with neutral evolution in advanced CRC. In this review, we introduce recent advances in the field of ITH including the general component of ITH, clonal selective factors that consolidate the evolutionary process, and a representative clinical application of ITH.

## 1 INTRODUCTION

In general, intratumor heterogeneity (ITH) is considered one of the critical causes of intractability in the treatment of cancers; therefore, it is very important to clarify the precise mechanism underlying ITH to establish a strategy for the treatment of solid cancers.

Recently, ITH‐related studies have used next‐generation sequencing to conduct whole‐exome sequencing of multiple excised samples from primary and/or metastatic tumors and have comprehensively integrated whole sequence data (Table 1).^1^, ^2^, ^3^, ^4^, ^5^, ^6^, ^7^, ^8^, ^9^, ^10^, ^11^, ^12^, ^13^, ^14^, ^15^, ^16^, ^17^, ^18^, ^19^, ^20^, ^21^, ^22^, ^23^, ^24^, ^25^, ^26^, ^27^, ^28^, ^29^, ^30^, ^31^, ^32^, ^33^, ^34^, ^35^ This multiregional analysis (MRA) sequencing approach enabled us not only to observe spatial heterogeneity, but also to calculate temporal alterations and eventually disclose the evolution of tumors. There are two types of somatic aberration in a tumor: ubiquitous aberrations (founder mutations, trunk mutations, or clonal mutations) and scattered aberrations (progressor mutations, branch/leaf mutations, or subclonal mutations). The former and the latter are triggered by a carcinogenic event and a late event, respectively.

**Table 1:** Achievements in the field of intratumor heterogeneity and evolutions of solid cancers

	Objectives	Journal	Year^Ref.^
Brain	425 Glioma from 54 cases	Nat Genet	2015^1^
	33 Medulloblastoma samples	Nature	2016^2^
	114 Cases of glioblastoma	Nat Genet	2016^3^
Breast	100 Cells from 2 cases	Nature	2011^4^
	303 Samples from 50 cases	Nat Med	2015^5^
	1000 Single cells from 12 cases	Nat Genet	2016^6^
	3 ER+HER2‐, 1 TN	PLoS Med	2016^7^
	10 Autopsied cases	Nat Commun	2017^8^
Colon	349 Glands from 15 cases	Nat Genet	2015^9^
	306 Polyps (6–9 mm)	Gut	2017^10^
	75 Samples from 10 cases	PLoS Genet	2016^11^
Esophagus	40 Samples from 8 cases	Cancer Discov	2015^12^
	25 Barrett's from 5 cases	Nat Genet	2015^13^
	51 Samples from 13 cases	Nat Genet	2016^14^
Head and neck	1 HNC and 2 nodes	Neoplasia	2013^15^
Liver	23 Cases of HCC	Proc Natl Acad Sci USA	2015^16^
	120 Samples from 23 cases	Clin Cancer Res	2015^17^
	43 Samples from 10 HCC	Gastroenterology	2016^18^
Lung	25 Samples from 7 NSCLC	Science	2014^19^
	11 Lung adenocarcinomas	Science	2014^20^
	100 From the TRACERx cohort	Nature	2017^21^
	100 Early‐stage NSCLC	N Engl J Med	2017^22^
Melanoma	41 biopsies from 8 cases	Cancer Res	2016^23^
Ovary	135 Samples from 14 cases	PLoS Med	2015^24^
Pancreas	7 Autopsies	Nature	2010^25^
	214 Samples	Nature	2016^26^
Prostate	7 Distant metastases	J Clin Invest	2013^27^
	57 Tumors	Cell	2013^28^
	5 Cases for methylation	Cell Rep	2014^29^
	10 Cases for resistant to TX	Nature	2015^30^
Kidney	9 Samples from 1 case	N Engl J Med	2012^31^
	10 Cases for signature	Nat Genet	2014^32^
Urothelium	72 Samples from 16 cases	Nat Genet	2016^33^

In addition, we disclose the clinical significance of defining the clonality of genomic aberrations by the MRA method from the viewpoint of targeting the cancers. In the phylogenic tree of ITH in the study of cancer evolution, clonal mutations were located in the trunk, and minor mutations were in branches and leaves. According to Willyard et al,^34^ to identify a bona‐fide target in ITH providing sufficient antitumor effect, we should target any clonal events in the trunk for eliminating cancer.

In this review, we update the general information of ITH as follows: (i) components of ITH; (ii) evolution model for chronological factors for ITH; (iii) clonal selective factors for fostering ITH; and (iv) a representative large study of the clinical applications of ITH.

## 2 GENERAL COMPONENTS OF ITH

### 2.1 Mutation spectrum

The degree of temporal and spatial heterogeneity depends on the malignancy. In general, melanoma and lung cancer accumulated a large number of somatic nucleotide variants (SNV); however, the diversity level was low and SNV were relatively ubiquitous in the tumor. Somatic mutations are present in all cells and they are the consequence of multiple mutational processes, including the intrinsic slight infidelity of the DNA replication machinery, exogenous or endogenous mutagen exposures, and enzymatic modification of DNA and, at present, they have been classified into 30 signatures.^35^, ^36^, ^37^, ^38^ The nucleotide substitutions in those tumors were characterized as C>T from ultraviolet light (signature 7)^23^ and C>A from smoking (signature 4).^19^, ^20^ Both substitutions were observed as founder events. These findings indicated that strong outer mutagens, such as UV light and cigarettes, may be carcinogens. In addition, low‐grade glioma showed an exacerbated diversity in ITH after treatment with the alkylating agent temozolomide.^39^ In non‐small‐cell lung cancers (NSCLC) and bladder cancer, the apolipoprotein B mRNA editing enzyme, catalytic polypeptide‐like (APOBEC) family of proteins was associated with fostering many subclones (signature 2).^19^, ^20^, ^40^

### 2.2 Causative mechanism of ITH

There is a dispute between natural (Darwinian) selection and neutral evolution. In renal cell carcinoma, driver genes, such as *mTOR*,* TSC1*,* PTEN*, and *PIK3CA*, were observed at subclonal or parallel positions in an identical tumor.^31^, ^32^ These alterations were considered to be a result of natural selection. On the contrary, progressor alterations accumulated in few driver genes, and the passenger mutations fostered neutral evolution. According to the distribution of variant allele frequency (VAF), neutral evolution was observed in 30% of all malignancies.^41^ Our previous whole‐exome sequencing (WES) study by MRA showed neutral evolution in advanced CRC cases, which was validated by computational simulation analysis. According to our previous model, accumulation of non‐driver genes, presence of cancer stem cells, and the microenvironment around cancer cells can foster neutral evolution in advanced CRC.^11^

## 3 EVOLUTION MODEL AND CHRONOLOGICAL FACTORS TO FORM ITH

**Figure 1:**
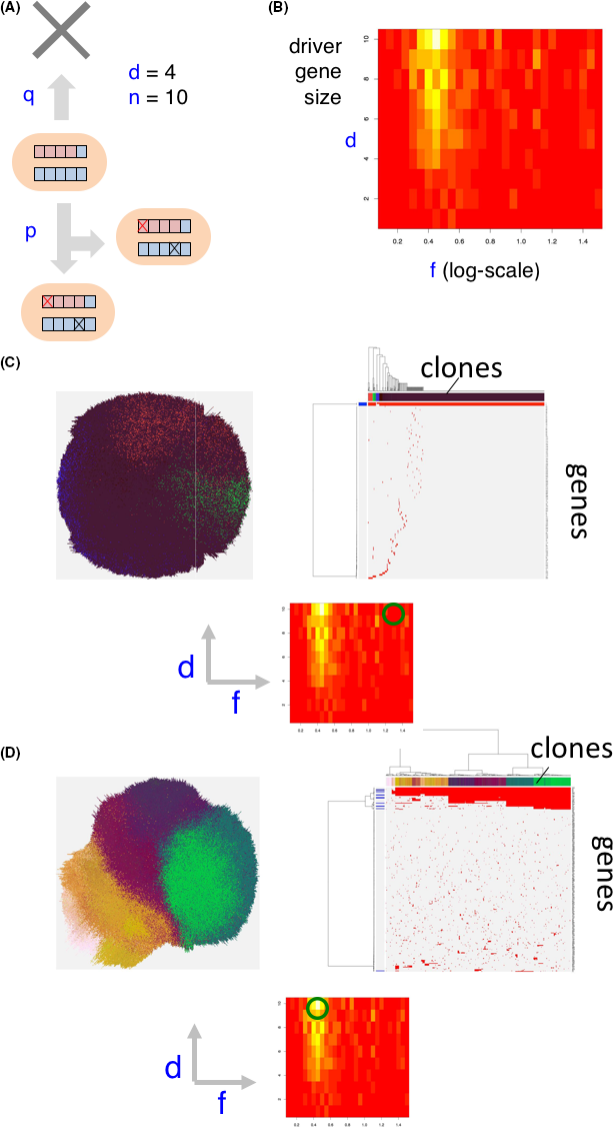
Branching evolutionary process (BEP) model. A, A cell has n genes, d of which are driver genes. In a unit time step, a cell divides and dies with probabilities p and q, respectively. A cell division mutates each gene with a probability r. One driver mutation increases p by f‐fold. In this model, f indicates strength of the driver genes. B, Population entropy depends on parameters d and f. The division probability increases per driver mutation. Red area indicates negentropy or syntropy, whereas white area indicates entropy. C, Existence of strong driver genes leads to a homogenous tumor. D, Multiple driver genes of moderate strength generate intratumor heterogeneity

Our multiregional sequencing study showed that progressor mutations comprised 40% of all mutations, and most of them were classified as passenger mutations and form ITH. Neutral evolution along with clonal evolution is a principal cause of ITH and fosters advanced CRC.^11^ We simulated heterogeneous cancer evolution as “branching evolutionary process (BEP) model” by supercomupter (Figure 1). In this model, each cell gradually accumulates driver mutations as well as accompanying passenger mutations, which do not affect the cell division rate and, finally, a tumor is formed with numerous accumulated mutations. According to the model, we found that mutations in driver genes were clonal, and non‐driver genes were subclonal; therefore, advanced CRC showed ITH not by natural selection, but by neutral evolution. From the viewpoint of the early phase of evolution, Sottoriva et al^9^ reported that clonal expansions or selective sweeps are extremely rare after the transition to an advanced tumor as a result of the dynamics and spatial constraints of the rapidly growing population. They proposed a “Big Bang” model as a result of single clonal expansion in which the most detectable ITH occurs at a very early phase after the transition to an advanced tumor, and then these subclones expand without natural selection, while partially mixing, to eventually show uniformly high ITH in every region of the tumor.

## 4 CLONAL SELECTIVE FACTORS FOR FOSTERING ITH

### 4.1 Chemotherapy and treatment

**Figure 2:**
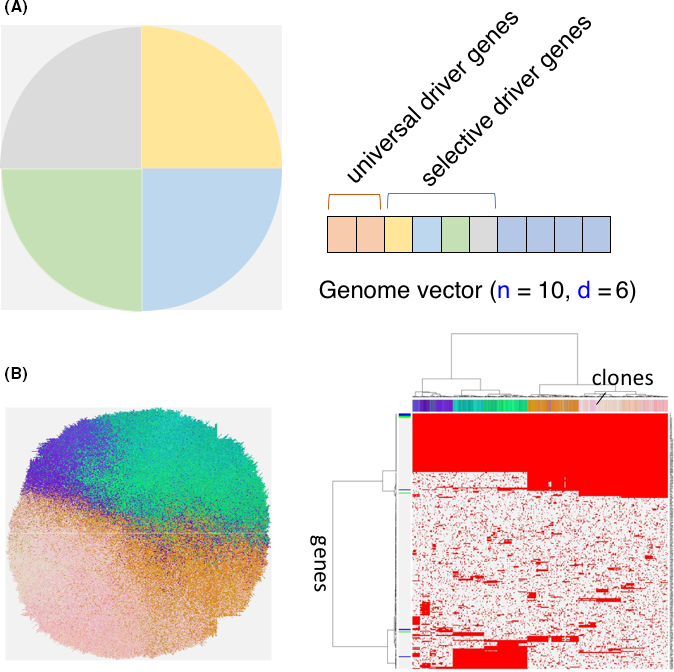
A, Implementation of environmental selection (n, number of genes; d, number of driver genes). If mutation has occurred in each quadrant of the tumor, selective driver genes increase growth rate. B, Existence of environmental selection can also enhance intratumor heterogeneity, which looks close to the actual heterogenous tumor

According to the MRA of bladder cancer and ovarian cancer, there were several cases in which anticancer drugs and hormonal agents might determine the clones that survive. Treatment of cancer might be one of the selective pressures on clones, and recurrence might be derived from surviving clones.^7^, ^33^ As depicted in Figure 2, we implemented the simulation study to prove the presence of ITH with selective mutations in driver genes by exposing four environmental pressures, such as chemotherapy and other treatment modalities. Existence of environmental selection can also enhance intratumor heterogeneity which is shaping the real heterogenous tumor.

### 4.2 Anatomical microenvironment

In general, clones with diverse ITH acquired advantages for sustained survival. However, the cancer microenvironment, such as the blood flow, oxygen level, and anatomical structures, could be selective pressures for clones to survive. Considering the fate of cancer cells, a malignant cell will meet and surpass various pressures during cancer progression. According to a mouse model, recurrence of a primary tumor demands multiple propensities for each clone, such as invasion, local dissemination, vascular embolus, circulating tumor cells, and micrometastasis.^42^ Therefore, ITH might provide advantages for metastasis.

### 4.3 Cytokines in the microenvironment

Non‐cell autonomous driving of tumor growth stabilizes subclonal heterogeneity, thereby enabling the survival of interclonal interactions. A xenograft model of chemokine‐producing transgenic mouse‐derived clones and parental clones was applied in a study by Marusyk et al.^43^ In terms of the variability between the groups in morphology, proliferation, and vascularization, only chemokine (C‐C motif) ligand 5 (CCL5)‐ and interleukin 11 (IL‐11)‐overexpressing subclones were able to enhance tumor growth. Tumor progression is frequently limited by microenvironmental constraints that cannot be overcome by the autonomous increase in cell proliferation rates. Instead, progression depends on alterations of the microenvironment, accelerated by cytokines.^43^

### 4.4 Clonal selection by dN/dS ratio and purifying selection and positive selection

Primary advanced cancer consists of numerous neutral evolutions with few driver genes. Considering the treatment of cancer, we have to confront the difficulties in eradicating cancers with many neutral mutations. We focused on the selection of clones as a result of mutations. Nucleotide substitutions in genes coding for proteins can be classified as either synonymous (does not change the amino acid) or non‐synonymous (changes the amino acid). dN/dS is the ratio between the rate of non‐synonymous substitutions per non‐synonymous site and the rate of synonymous substitutions per synonymous site.^44^, ^45^ In general, most non‐synonymous changes would be expected to be eliminated by purifying selection, but, under certain conditions, natural selection may lead to their retention. Investigating the number of synonymous and non‐synonymous substitutions may, therefore, provide information about the degree of selection of genes.^46^

Clones carrying passenger mutations would senesce or die, such that the mutation would be lost from the catalog of variants seen in resected cancer specimens. This is negative or purifying selection, which leads to a dN/dS <1 in a given gene or set of genes, if it occurs at appreciable rates.^47^, ^48^, ^49^, ^50^ If the negatively selected genes are 0.02%‐0.5% of all genes, the dN/dS <1, and clones with coding mutations will be lost per tumor. On the contrary, some somatic mutations, such as driver genes, can confer a growth advantage, whereas others may impair cell survival or proliferation. Positively selected genes have a dN/dS ≥1 and were 1%‐3.9% of all genes. There were 1‐10^+^ driver mutations per tumor, and they had a much stronger force than negative selection. According to the study by Martincorena et al,^50^ CRC showed a relatively higher dN/dS ratio than other cancers. Therefore, the dN/dS ratio indicated that positively selected genes might exist under negative selection in advanced CRC. However, our previous study disclosed neutral evolution in advanced CRC with a number of minor (non‐driver) mutations according to our multisampling and sequence data.^11^ To comprehend this contradiction, we have provided the following possible explanation.

### 4.5 Impaired neoantigen presentation by chromosomal aberration

Immune evasion is one of the hallmarks of cancer. Losing the ability to present neoantigens, as a result of human leukocyte antigen (HLA) loss, may facilitate immune evasion. McGranahan et al^40^ reported that HLA loss of heterozygosity (LOH) occurs in 40% of NSCLC. LOH is an immune escape mechanism that is subject to strong microenvironment later in tumor evolution. Subclonal LOH was observed on chromosome 6, which harbored HLA haplotypes, and the number of putative neoantigens presented to T cells was clearly impaired.^42^ Therefore, cells with a high number of somatic mutations might not present neoantigens and evade the immune response.

In addition, compared with early CRC, copy number aberrations were specifically observed in advanced CRC (T. Saito, unpublished data). The number of somatic mutations on chromosomes was drastically altered, along with aneuploidy or chromosomal loss in advanced CRC. Many non‐driver, passenger genes on the amplified chromosomes might not have been eliminated and were not affected by natural selection. Further studies will be required to prove it.

## 5 FURTHER ANALYSIS OF ITH FOR CLINICAL APPLICATIONS

To prospectively investigate ITH in relation to clinical outcome and to determine the clonal nature of driver events and evolutionary processes in early‐stage NSCLC, Jamal‐Hanjani et al^22^ sequenced 327 tumor regions and 100 matched germline samples derived from whole blood. These data may have important implications for understanding tumor biology and therapeutic control in NSCLC. This project is called Tracking Cancer Evolution through Treatment (TRACERx).

## 6 CONCLUSION

Multiregional sequencing analysis of solid clinical samples provides a major breakthrough in disclosing ITH. The level of uniformity depends on the type of cancer, and the causes of diversity vary among cancers. On the contrary, in vivo analysis showed that heterogeneity sustains mutually surviving cancer cells in a cluster and provides an advantage for metastasis. Actual application of the findings in the present review for clinical diagnosis and treatment might require more time to save patients from intractable cancers.

## DISCLOSURE

There is no relevant financial or nonfinancial relationships to disclose.
